# Low-dose morphine and airway sensations in refractory chronic cough: a randomised control trial

**DOI:** 10.1183/23120541.00173-2026

**Published:** 2026-09-01

**Authors:** Bashar D. Al-Sheklly, Joanne Mitchell, Kimberley Holt, Rachel Dockry, Huda Badri, Imran Satia, Terri Collier, Shilpi Sen, Jaclyn A. Smith

**Affiliations:** 1 Manchester University NHS Foundation Trust, Manchester, UK; 2 Division of Infection, Immunity and Respiratory Medicine, University of Manchester and Manchester Academic Health Science Centre, Manchester, UK; 3 Department of Medicine, McMaster University, Hamilton, ON, Canada

## Abstract

**Background:**

Low-dose morphine is recommended as an antitussive agent for refractory chronic cough (RCC), although its precise mechanism of action remains unknown. We hypothesised that noxious airway sensations (*e.g.* throat irritation, tickle, urge to cough) described by RCC patients play a role in initiating coughing and are lessened by morphine.

**Methods:**

22 RCC patients (median age 65 years; 83% female) reporting a clinical response to low-dose morphine withdrew from therapy and enrolled in a double-blinded, randomised, placebo-controlled, crossover trial comparing the effects of their usual dose of morphine with those of a matched placebo. The sensations evoked by citric acid inhalation were assessed at baseline and at the end of each 5–7-day treatment period. The efficacy of morphine, compared with placebo, was assessed by objective cough monitoring and patient-reported outcomes at the same time points.

**Findings:**

Compared with placebo, low-dose morphine improved the rating of all sensations provoked by citric acid inhalation (all p<0.05), but the cough threshold (C2) was not changed (p=0.61). 24-hour cough frequency was reduced by 71.8% with low-dose morphine, compared with placebo treatment (p<0.001), and all patient-reported outcomes significantly improved.

**Interpretation:**

This trial suggests that the antitussive action of low-dose morphine may be a consequence of modulation of noxious airway sensations and provides the first objective evidence of its efficacy in RCC patients reporting a clinical response.

## Shareable abstract

**Noxious airway sensations are associated with the initiation of coughing, *i.e.* tickle, irritation and the urge to cough. Low-dose morphine significantly reduced both these sensations and cough frequency in a refractory chronic cough responder group.** https://bit.ly/4tPO59A

## Introduction

Chronic cough (>8 weeks’ duration) affects up to 12% of the population, with patients coughing hundreds and up to thousands of times a day [1, 2]. Physical, social and psychological problems are reported, significantly impairing quality of life [3]. Refractory chronic cough (RCC) is a condition that is unresponsive to standard treatment approaches for gastro-oesophageal reflux, nasal disease and asthma, and when no underlying condition is identified [4, 5]. Although novel treatments are in development, the only new therapy for RCC is not licensed in all countries and access is limited even when licensed. Low-dose morphine has been shown to improve cough-specific quality of life for patients with this condition, and it is recommended by guidelines [5–7].

Animal studies show that specific sensory vagal afferent nerves are responsible for evoking cough and relay information from the airways to the brainstem [8]. Functional brain imaging studies in healthy humans have found widely distributed central nervous system activation associated with the urge-to-cough sensations evoked by inhaled capsaicin (*e.g.* primary and secondary sensory cortices, insula and cingulate cortex) [9]. RCC is increasingly believed to result from a hyperexcitability of these peripheral and/or central pathways. Consistent with this notion, in a similar study, RCC patients exhibited increased activity in the midbrain on capsaicin exposure, including the periaqueductal grey (PAG), when compared with healthy controls [10].

Opioids have long been considered effective antitussive agents, although their precise mechanism of action remains unknown and robust clinical trial data to support their use are scant. A single randomised controlled trial of low-dose, slow-release morphine sulfate in RCC patients reported that morphine significantly improved cough patient-reported outcomes (PROs), compared with placebo; however, cough responses to inhaled citric acid were unchanged and cough frequency was not objectively measured [6]. Opioid receptors are present in the lungs as well as the central nervous system [11]. In the brain, high concentrations of opioid receptors are found in the PAG, thought to be important in opioid-induced analgesia. Stimulation of this area activates descending pathways, inhibiting the transmission of painful sensations to the brain. Chronic cough patients report a variety of sensations preceding coughing, most commonly throat irritation and tickle, and associate these with an urge to cough [12, 13]. Therefore, it is conceivable that morphine inhibits noxious airway sensations in a similar manner, thereby alleviating coughing.

We aimed to investigate the effect of low-dose morphine on the airway sensations associated with coughing in patients with RCC. We used a modified citric acid challenge protocol to assess the sensations experienced prior to the initiation of coughing. We recruited RCC patients taking low-dose morphine as a clinical treatment for their cough to a randomised, double-blind, placebo-controlled, two-way crossover single-site trial. The antitussive effect of low-dose morphine was evaluated by objective cough monitoring and PROs.

## Methods

### Participants

We recruited RCC patients from a specialist cough clinic (Manchester University NHS Foundation Trust, Manchester, UK) who had been diagnosed in accordance with current guidance and who were taking, and reported benefit from, 5–10 mg of slow-release morphine twice daily [14]. Current and ex-smokers with a >20 pack-year history were ineligible, as were those with conditions that could modify cough (*e.g.* Parkinson's disease, stroke) or pose safety risks. Those on other treatments that modulate (*e.g.* gabapentin) or potentiate (*e.g.* angiotensin-converting enzyme inhibitors) cough were excluded, along with those with respiratory tract infections within the 4 weeks prior to screening. Pregnant or breastfeeding women were also excluded.

### Trial design

**FIGURE 1:**
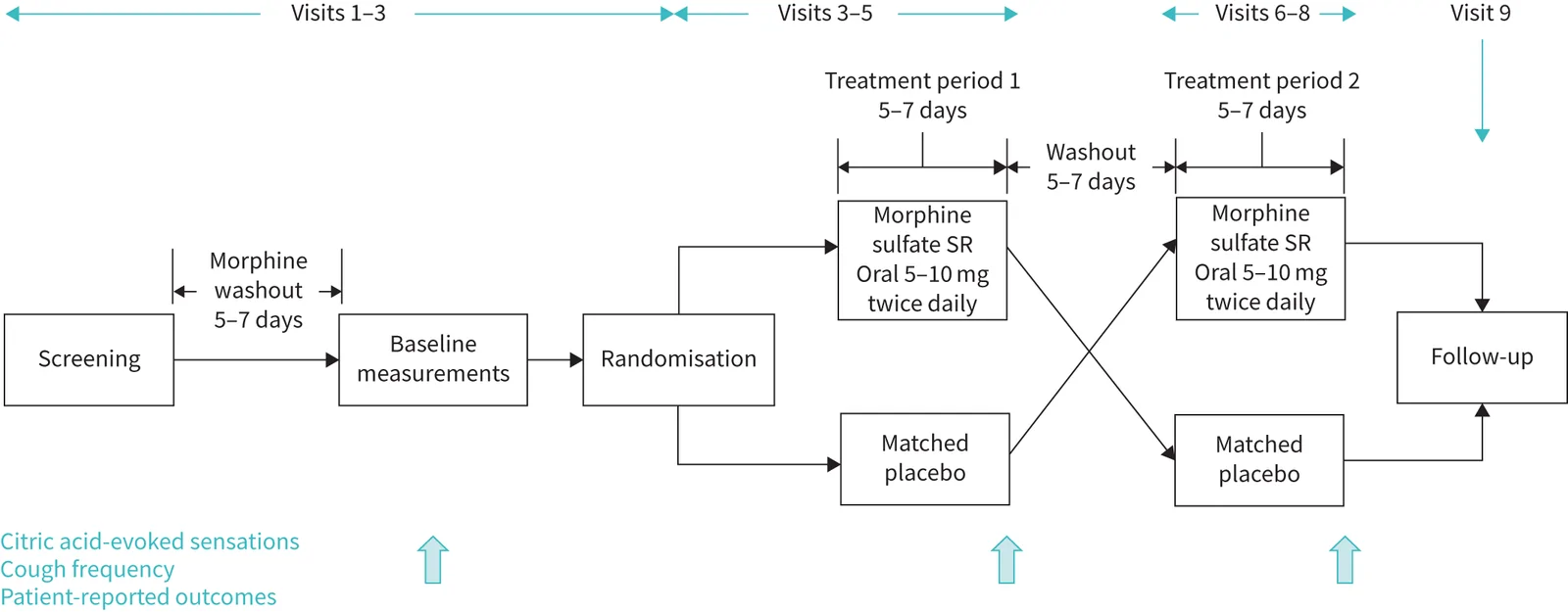
Trial design. SR: slow release.

We performed a double-blind, randomised, placebo-controlled, crossover trial to evaluate the effects of low-dose, slow-release morphine sulfate on airway sensations thought to initiate cough. On enrolment, patients withdrew from their usual morphine therapy; after a 5–7 day washout, they were randomised to receive morphine (matched to their pre-trial dose) followed by a matched placebo, or *vice versa*, during two treatment periods (5–7 days’ duration) separated by a 5–7 day washout (figure 1). Airway sensations were assessed by a modified citric acid challenge at baseline and at the end of each treatment period. Antitussive efficacy was evaluated by 24-hour ambulatory cough monitoring and PROs at the same time points. The trial was approved by an independent Research Ethics Committee (15/NW/0153). Participants gave written informed consent and were recruited between December 2015 and March 2017.

### Trial treatment and randomisation

Participants were assigned to one of two possible treatment sequences by a computer-generated randomisation schedule with a ratio of 1:1 for morphine/placebo or placebo/morphine. Both placebo and morphine (5 mg) were identically encapsulated and provided in blinded packaging to the hospital pharmacy for dispensing (Sharp Clinical Services, Powys, UK). Subjects previously taking 10 mg of morphine sulfate twice daily took two capsules twice daily, and those on 5 mg twice daily took one. Due to the double-blind nature of the trial, neither participants nor research staff were aware of a participant's treatment sequence.

### Procedures

Participant demographics, medications and comorbidities were collected at the first study visit and eligibility checked. Safety was assessed through monitoring of vital signs, spirometry before and after the cough challenge and adverse event recording. Urine was tested for opioids at the start and end of each treatment period to check compliance; these results were not reported to the study team until unblinding.

#### Cough challenges

Following an inhalation of 0.9% saline, single breaths of increasing doses of citric acid (0.01, 0.015, 0.02, 0.03, 0.04, 0.06, 0.09, 0.125, 0.18, 0.25, 0.375, 0.5, 0.75, 1.0, 1.5, 2, 3, 4 M) were inhaled at 1-minute intervals from a calibrated nebuliser chamber (DeVilbiss Healthcare, Somerset, PA, USA) attached to a dosimeter (KoKo Dosimeter, Ferraris, Hertford, UK) with flow limitation. Participants were asked to complete four 100-mm visual analogue scales (VASs) after each inhalation to document the intensity of sensations evoked (see supplementary material for details). In our previous qualitative studies, we found that patients with chronic cough most commonly describe the sensation(s) associated with coughing as a throat irritation (86%) and/or throat tickle (73%); fewer (69%) reported an urge to cough prior to coughing. We therefore asked participants to rate these sensations in response to the citric acid exposure; taste was also assessed [12, 15, 16]. The number of coughs evoked in 15 s following each inhalation was counted. The challenge was completed when the C2 dose was achieved (*i.e.* the lowest dose evoking at least two coughs in the 15 s after inhalation) or if all inhalations were completed without cough occurring.

#### Ambulatory cough monitoring

24-hour acoustic recordings were made using the VitaloJAK™ cough monitor (Vitalograph Ltd, Buckinghamshire, UK), with cough sounds per hour quantified by a semi-automated method using validated custom-written software [17]. This was performed at baseline (prior to washout), following washout and once at the end of each treatment period.

#### Patient-reported cough measures

Subjects rated cough severity on a 100-mm VAS and numerically (from 0–5) for both awake and sleep periods at baseline, following washout and on the last day of each treatment period. Cough-specific quality of life was assessed prior to and during each treatment period using the Cough-specific Quality of Life Questionnaire (CQLQ); a higher score indicates a worse quality of life [18].

#### Questionnaires

Participants also completed the Somatosensory Amplification Scale [19] once, at baseline, and the State–Trait Anxiety Inventory (STAI) [20] at baseline and following washout.

### Outcomes

The primary outcome was the effect of low-dose morphine, compared with placebo, on the intensity of airway sensations (throat irritation, tickle, urge to cough) and taste evoked by citric acid inhalation. Secondary outcomes examined the efficacy of low-dose morphine in reducing objective cough frequency and PROs. At baseline (after morphine washout), the relationships between sensations, urge to cough and cough initiation were also explored and any influence of somatosensory amplification and state–trait anxiety was assessed.

### Data analysis

SPSS (version 23, IBM Inc.) was used for data analysis. Airway sensations were plotted against citric acid concentration (stimulus strength) and aligned to individual C2 concentration (cough initiation). To assess correlations between sensations and urge to cough, within-subject Bland–Altman correlation analyses were performed. As the C2 varied between participants and not all subjects inhaled all citric acid concentrations, different numbers of ratings prior to coughing were available. Therefore, for these analyses only, ratings were analysed only when available for ≥50% of subjects. The relationship between changes in sensations and the initiation of coughing was evaluated using the Wilcoxon test to compare the rate of change of sensations from the beginning of the challenge to the initiation of coughing, measured in mm·M^−1^ (see supplementary material for details).

Linear mixed-effects modelling compared sensations at increasing concentrations of citric acid during morphine and placebo treatments, producing an estimated marginal mean for each sensation at each concentration of citric acid. All available data were included, a random slope was used and the models were adjusted for baseline measurements, period and sequence. Generalised estimating equation (GEE) models compared the effect of morphine *versus* placebo on efficacy endpoints. All GEE models were adjusted for baseline measurements, and the effects of period and sequence assessed. Both sensation ratings and cough frequency data were not normally distributed; therefore, they were log transformed for analysis. The standard p<0.05 level of significance was applied.

A power calculation (PS Power and Sample Size, Vanderbilt University) based on the between-subject standard deviation of the urge-to-cough VAS at baseline in the first 12 patients enrolled suggested that a sample size of 22 would be sufficient to detect a 20-mm difference in the urge-to-cough rating at C2 for morphine, compared with placebo (power 90%, 5% significance level).

## Results

### Subjects

**FIGURE 2:**
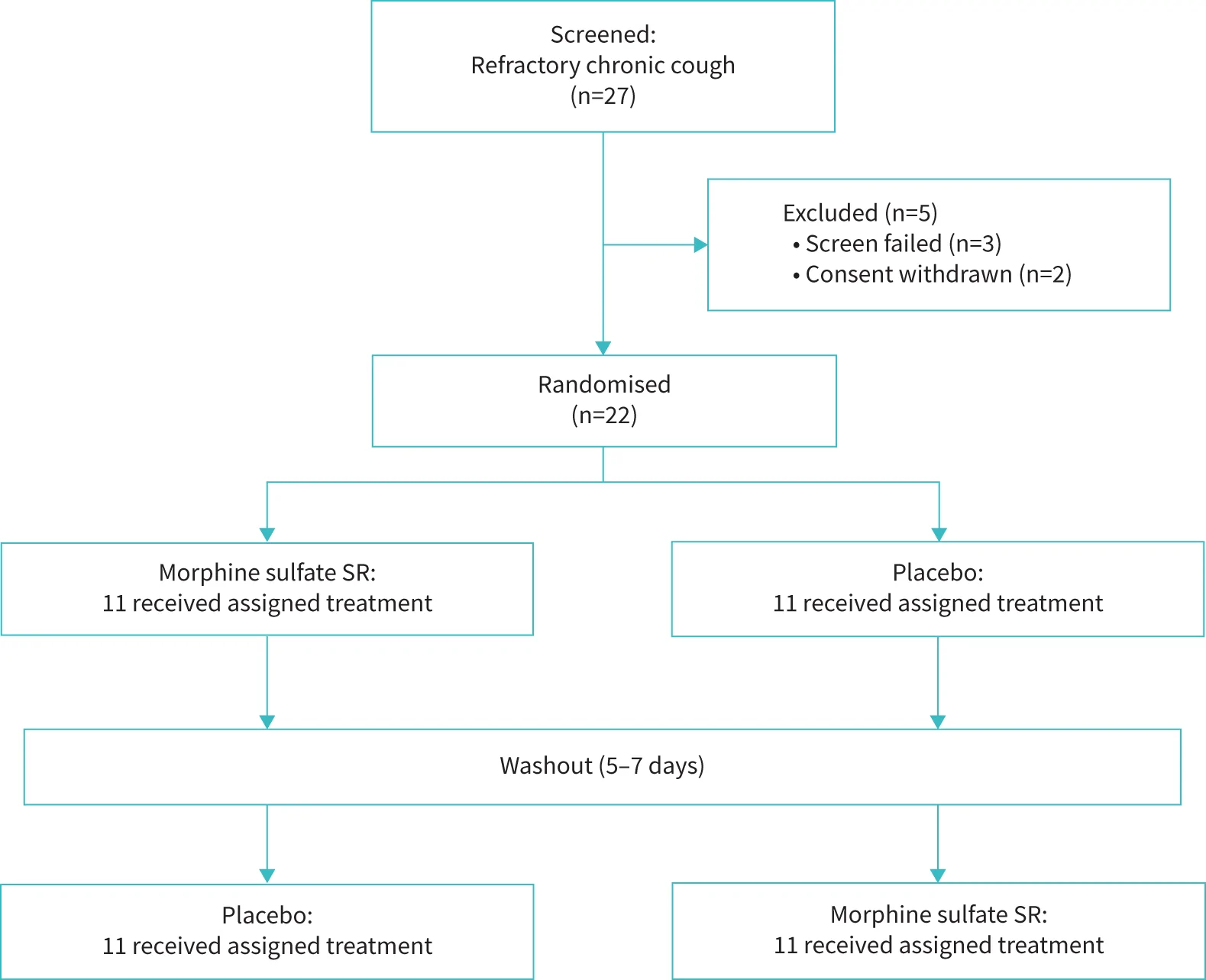
Consolidated Standards of Reporting Trials (CONSORT)#diagram. SR: slow release.

Of 27 chronic cough patients recruited, three failed eligibility screening and two withdrew consent prior to randomisation due to worsening of cough on discontinuation of morphine. A total of 22 subjects (median age 65 years, 18 female) participated in the study (table 1 and figure 2). Prior to enrolment, 18 were taking 5 mg of slow-release morphine sulfate twice daily and four took 10 mg twice daily.

**TABLE 1:** Characteristics of participants

**Demographic**	Refractory chronic cough patients (n=22)
**Age, years, median (IQR)**	65.0 (56.5–68.0)
**Sex, n (%)**	
Male	6 (18)
Female	18 (82)
**Height (cm)**	164.0 (7.3)
**Ethnicity: Caucasian, n (%)**	22 (85)
**Weight, kg, median (IQR)**	75.8 (61.6–85.2)
**BMI, kg·m^−2^**	27.6 (5.9)
**Smoking status, n (%)**	
Ex-smoker	6 (27)
Never-smoker	16 (73)
**Smoking pack-years, median (IQR)**	0 (0–1)
**FEV_1_ % predicted**	97.7 (15.3)
**FVC % predicted**	110.0 (15.7)
**FEV_1_/FVC ratio, median (IQR)**	74.5 (70.5–78.5)

Data are presented as mean±sd unless otherwise stated. IQR: interquartile range; BMI: body mass index; FEV_1_: forced expiratory volume in 1 s; FVC: forced vital capacity.

### Experimentally evoked sensations and cough at baseline

**FIGURE 3:**
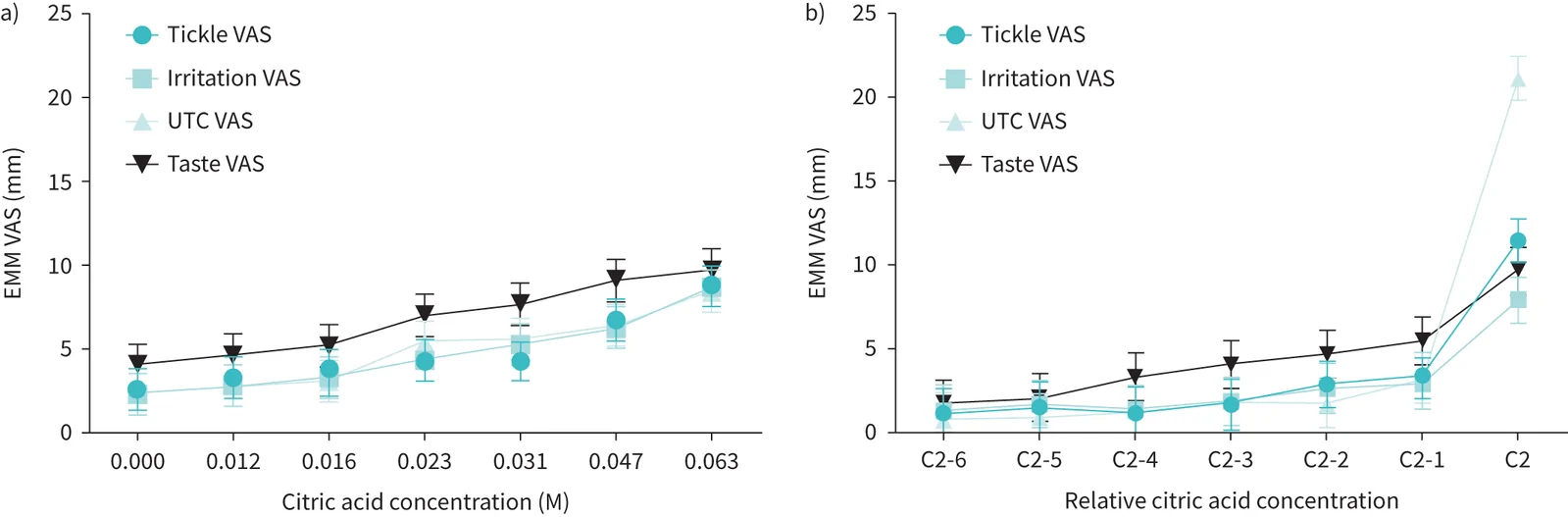
Sensations evoked by citric acid at baseline in participants with refractory chronic cough (after morphine washout). Data shown are estimated marginal mean (EMM)±sem visual analogue scale (VAS) scores for a) citric acid concentrations inhaled and b) concentrations of citric acid arranged by cough threshold (C2), *i.e.* the lowest concentration evoking at least two coughs and the preceding six concentrations. Only concentrations inhaled by at least 50% of subjects were included. UTC: urge to cough.

All RCC patients completed the modified citric acid challenge at baseline, *i.e.* following discontinuation of usual morphine therapy. The median C2 in chronic cough patients was 0.055 M (interquartile range (IQR) 0.021–0.125 M). There was no clear threshold below which no sensations were reported. When arranged by citric acid concentration, VAS scores for all sensations significantly increased with rising concentrations, up to the median concentration inhaled (p<0.001), with taste rated slightly more highly than other sensations by approximately 2–3 mm (figure 3a). When arranged by C2, taste was also rated marginally higher than other sensations until C2 was reached (figure 3b). At C2, *i.e.* when coughing started, the urge to cough was the most highly rated sensation. Rates of change in urge to cough, tickle, irritation and taste of the citric acid all increased significantly at initiation of coughing (p<0.05), with urge to cough the most strongly associated and taste the least (supplementary table E1). Sensations of tickle, irritation and taste all strongly correlated with the urge to cough (p<0.001) (supplementary figure E1). Somatosensory amplification and STAI state–trait anxiety scores did not correlate with VAS sensation scores when corrected for multiple testing (supplementary tables E2 and E3).

### Effect of morphine on experimentally evoked sensations

**FIGURE 4:**
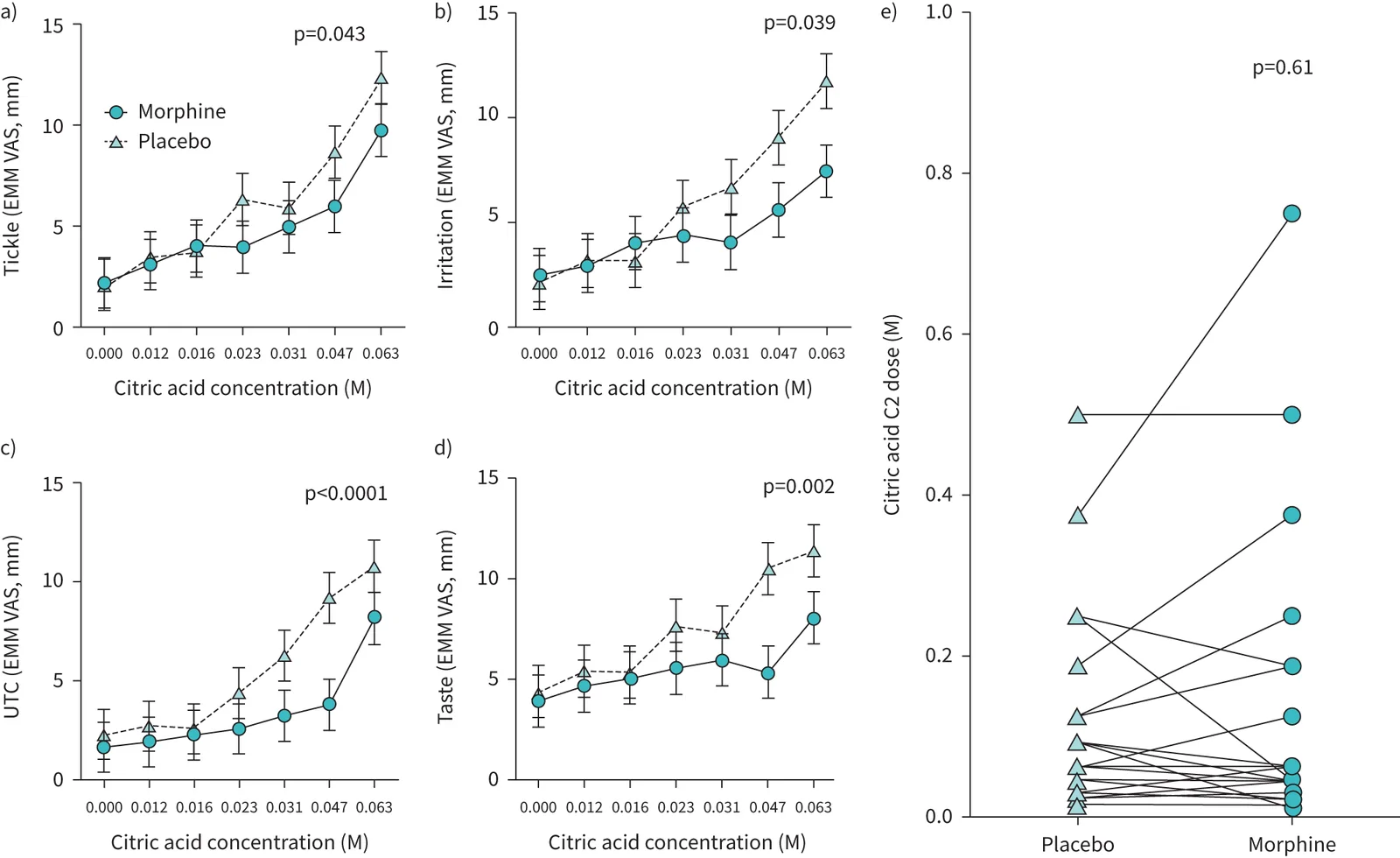
Effect of morphine sulfate, compared with placebo, on sensations and cough evoked by citric acid inhalation in participants with refractory chronic cough who report a clinical response to morphine. Data shown in a–d) are estimated marginal mean (EMM)±sem visual analogue scale (VAS) scores from the linear mixed-effects model, plotted against citric acid concentration inhaled. We only included concentrations that were achieved by at least 50% of subjects in both groups. a) Tickle against citric acid concentration; b) irritation against citric acid concentration; c) UTC against citric acid concentration; d) taste against citric acid concentration; e) the effect of citric acid on C2. UTC: urge to cough; C2: cough threshold.

When the results were arranged by citric acid concentration, low-dose slow-release morphine sulfate was associated with a statistically significant reduction in VAS ratings for all four experimentally evoked sensations, compared with placebo (all p<0.05) (figure 4a–d). When sensation scores were arranged by C2, morphine treatment significantly reduced the VAS scores only for urge to cough and taste of the citric acid (supplementary figure E2). Of note, there was no significant difference in the C2 concentration between morphine and placebo (p=0.61) (figure 4e).

### Antitussive efficacy measurements

#### Cough frequency

**FIGURE 5:**
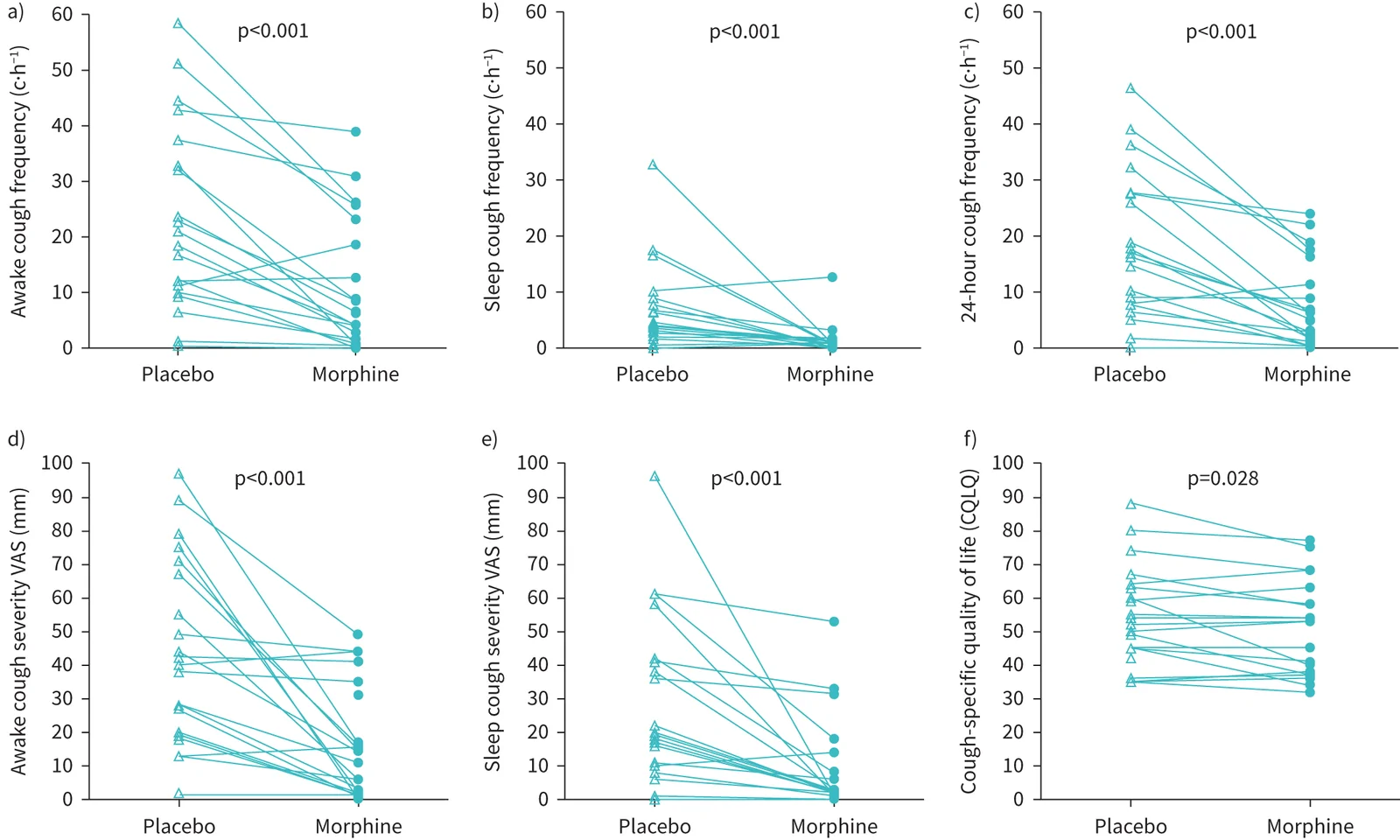
Efficacy of morphine, compared with placebo, in morphine responders with refractory chronic cough (p<0.05 for all). a) Awake cough frequency; b) sleep cough frequency; c) 24-hour cough frequency; d) awake cough severity VAS; e) sleep cough severity VAS; f) cough-specific quality of life, measured with the CQLQ. c·h^−1^: coughs per hour; VAS: visual analogue scale; CQLQ: Cough-specific Quality of Life Questionnaire.

22 participants completed baseline 24-hour cough monitoring (following morphine washout). 21 subjects completed 24-hour recordings after both morphine treatment and placebo treatment; one recording was missing after morphine treatment due to a recording failure, and one was missing after placebo treatment due to scheduling conflict. Low-dose morphine significantly reduced cough frequency compared with placebo: a 71.8% reduction over 24 h with no significant period or sequence effects (p<0.001) (table 2). The treatment effect was independent of baseline cough frequency (p=0.32). Similar results were seen for cough frequency divided into awake and sleep periods (figure 5a–c).

**TABLE 2:** Results of efficacy outcomes in the randomised controlled trial, with p-values, comparing morphine and placebo from the generalised estimating equation analysis

Endpoint	Baseline	Treatment		Difference (morphine *versus* placebo)	p-value
		Placebo	Morphine		
**Cough frequency, c·h^−1^, geometric mean (95% CI)**					
Awake	23.0 (9.8–41.0) (n=22)	21.0 (10.7–40.1) (n=21)	6.3 (1.3–20.9) (n=21)	−72.6%	**<0.001**
Sleep	4.8 (1.53–11.75) (n=22)	4.2 (2.2–9.6) (n=21)	0.7 (0.0–1.5) (n=21)	−91.4%	**<0.001**
24 hours	17.9 (9.2–30.4) (n=22)	17.0 (7.8–30.0) (n=21)	5.3 (1.4–13.9) (n=21)	−71.8%	**<0.001**
**Cough severity VAS, mm**					
Awake	47.7±27.6 (n=22)	43.5±16.5 (n=21)	16.3±27.2 (n=22)	−28.0 mm	**<0.001**
Sleep, geometric mean (95% CI)	25.0 (7.5–59.8) (n=22)	19.0 (9.0–41.5) (n=21)	3.0 (1.8–9.5) (n=22)	−19.1 mm	**<0.001**
**CQLQ**	57.1±11.7 (n=19)	54.0±14.1 (n=21)	51.1±15.0 (n=20)	−3.5	**0.028**
**Cough numeric score (0–5)**					
Awake	2.9 (0.9) (n=22)	3.0 (1.0) (n=21)	1.6 (1.1) (n=22)	−1.5	**<0.001**
Sleep	2.0 (1.3) (n=22)	1.8 (1.1) (n=21)	0.9 (0.7) (n=22)	−0.9	**<0.001**
**Citric acid C2, M, geometric mean (95% CI)**	0.055 (0.016–0.125) (n=22)	00.063 (0.047–0.25) (n=22)	0.0785 (0.031–0.188) (n=22)	+0.007 M	0.61

Data are presented as mean±sd unless otherwise stated. c·h^−1^: coughs per hour; VAS: visual analogue scale; CQLQ: Cough-specific Quality of Life Questionnaire; C2: cough threshold.

#### Patient-reported outcomes

VAS and numeric cough severity scores for awake and sleep also significantly improved with morphine treatment, compared with placebo (all p<0.001), as did cough-specific quality of life (p=0.028) (table 2, figure 5d–f and supplementary figure E3). There were no period or sequence effects in these analyses, aside from a sequence effect for sleep cough severity VAS; this suggested that those taking placebo before morphine had lower baseline sleep VAS scores, but this had no influence on the treatment effect (p>0.05).

### Adherence, safety and adverse events

After unblinding, one participant had a positive opioid urine test following the placebo treatment period, although the subject denied opioid use. Excluding the results from this participant made no difference to the overall results. Adverse events are shown in table 3; the most frequently reported was worsening cough during placebo treatment. One serious adverse event occurred during the trial follow-up period, but was not thought to be related to the trial drug.

**TABLE 3:** Adverse events log for the randomised controlled trial split into trial periods

Adverse event	Morphine treatment period (n)	Placebo treatment period (n)
**Worsening cough**	0	4
**Back pain**	0	1
**Blocked nose**	0	1
**Fatigue**	1	0
**Restlessness and poor sleep**	0	1
**Joint pains**	1	0
**Diarrhoea and cramps**	0	0
**Nausea and vomiting**	1	0
**Arm pain**	1	0
**Cheek swelling**	1	0

## Discussion

Cough is considered the archetypal airway reflex; in health, it protects the airway from the intrusion of foreign bodies and chemical irritants. However, it is increasingly appreciated that, rather than a typical reflex response, cough in disease often occurs in response to sensations of airway irritation and a consciously perceived compulsion to cough that is subject to a degree of voluntary control [21]. To the best of our knowledge, this is the first study to evaluate the influence of a pharmacological intervention on a range of sensations associated with coughing in RCC patients. Using a modified cough challenge methodology, we were able to demonstrate that low-dose slow-release morphine significantly reduced all measured airway sensations and the urge to cough evoked by citric acid inhalation, compared with placebo. This contrasted with the effects on the citric acid cough threshold (C2), which was not significantly changed. Furthermore, we have also provided the first objective evidence that low-dose morphine has a substantial effect on cough frequency and PROs in RCC patients reporting a clinical response; this occurred within a week of commencing treatment.

Several studies have investigated the urge to cough evoked by inhaled capsaicin, and functional imaging studies have explored the neural correlates of this model [9, 10, 22]. However, investigation of the range of somatic sensations described by chronic cough patients, in particular the common complaints of throat irritation and tickle, have been few [12]. Our data show that these sensations were reported with similar intensity to the urge to cough and responded in the same way to the inhalation of citric acid of increasing concentrations. However, sensations were found to differ at the point that coughing was initiated (C2), with the urge to cough most highly rated. Of note, RCC patients reported awareness of the taste of the citric acid solutions, suggesting this as a potential confounder in inhalation challenge studies, although the association with citric acid concentration was the weakest for taste, compared with other sensations.

We chose citric acid, rather than capsaicin, for this study for several reasons. Firstly, capsaicin activates C fibres only in the airways, yet cough can also be evoked by Aδ fibres. Citric acid has the advantage of activating both sets of fibres thought to be important in evoking cough. Secondly, capsaicin specifically activates transient receptor potential vanilloid 1 (TRPV1) receptors, but two negative randomised controlled trials of TRPV1 antagonists have suggested that this mechanism of activating cough is redundant in RCC patients [23, 24]. Citric acid cough challenges are known to be reproducible [25]. We did not continue the challenge to measure the C5 (*i.e.* the lowest dose evoking at least five coughs in the 15 s after inhalation), as morphine has already been found to not change this endpoint and this would have resulted in a very long challenge test for patients [6].

Our data also showed that morphine significantly inhibits the sensations associated with citric acid inhalation, compared with placebo. Although the reductions were small in magnitude, so were the overall ratings (typically <10 mm on a 100 mm scale). Notably, morphine failed to modify the threshold at which coughing occurred (the C2), which replicates the findings of previous studies of morphine in RCC and the effect of codeine on capsaicin-evoked cough and urge to cough in healthy volunteers [6, 26]. Another study did suggest that morphine significantly inhibits capsaicin-evoked cough responses, but only when administered intravenously at a dose of 0.15 mg·kg^−1^ to healthy volunteers [27]. Collectively, these results suggest that low-dose morphine mainly exerts its antitussive effects in RCC by modulating airway sensations and the urge to cough, rather than by shifting the threshold at which irritant stimuli provoke coughing.

It is possible to speculate about the processes that could underlie these observations. In this study, citric acid inhalation evoked relatively minor irritant sensations and urge to cough, and yet small reductions in these were associated with substantial changes in cough frequency and improvements in PROs. Such findings are consistent with the notion that coughing may, to some extent, be a behavioural response, seeking to relieve these sensations, analogous to scratching an itch. This may be exaggerated in RCC by an inability to consciously resist coughing, and also reduced descending inhibitory control mechanisms [21, 28]. Opioids enhance descending inhibitory pain pathways (*e.g.* in the PAG and rostroventromedial medulla), reducing perceived pain sensations [29]. Likewise, in RCC opioids may reinforce impaired descending inhibitory controls.

It may be unexpected that the reduction in sensory scores with morphine treatment also included the ratings of taste. However, μ-opioid receptors are implicated in the regulation of food intake and taste palatability, and some evidence suggests that opioids play an inhibitory role in gustatory processing [30]. It is also possible that subjects poorly discriminated between the different sensations they reported, reporting all uniformly reduced.

We observed a pronounced antitussive effect of morphine in this RCC responder group, with considerable improvements in cough frequency and PROs occurring within a relatively short treatment period. This magnitude of response is comparable to results seen for novel antitussives, for example in the first crossover trial of gefapixant, a novel P2X3 antagonist, which reduced cough frequency in RCC patients by 75%, compared with placebo [31]. In later parallel group studies, significant placebo effects offset the reduction compared with placebo. However, the effect in our study will have been amplified by selecting patients reporting a clinical response. Nonetheless, these results are consistent with positive findings reported recently for low-dose slow-release morphine for chronic cough in patients with idiopathic pulmonary fibrosis [32] and a study of a κ-opioid receptor agonist and μ-antagonist in RCC [33].

This study has some limitations. Since our aim was to investigate the mechanism of action of morphine, we recruited only those patients reporting a clinical benefit; in our experience, this is approximately 50% of RCC patients in a tertiary setting [34, 35]. Therefore, the effect of morphine on sensations in those not reporting a clinical response remains unknown. It should also be acknowledged that it is difficult to be certain that no unblinding occurred, due to subtle adverse effects and the substantial reduction in cough experienced with morphine, compared with placebo treatment. Morphine can cause drowsiness and constipation, leading to unmasking, but using low doses over a short treatment period ensured the drug was well tolerated, with no differences in reported adverse events compared with placebo. Finally, at present there are no validated tools for specifically capturing the sensations associated with coughing, either during experimental exposures or for capturing the spontaneous sensations experienced by participants in clinical trials, although some questionnaires contain individual items about these [13]. The findings of this study suggest a need for such tools.

In conclusion, we have demonstrated strong associations between experimentally evoked airway sensations (tickle, irritation and taste) and the compulsion to cough. We have also shown that, in a group of RCC patients reporting a clinical response, low-dose morphine is highly effective at reducing cough frequency and noxious airway sensations when compared with placebo. These observations suggest inhibition of noxious airway sensations, perhaps through agonism of opioid receptors in the brain (*e.g.* PAG), as a plausible mechanism of action of morphine in RCC patients.

## Data Availability

Data outside what are reported in the article or its supplementary material is available on reasonable request from cough.research@manchester.ac.uk.

## Supplementary material

**Please note:** supplementary material is not edited by the Editorial Office, and is uploaded as it has been supplied by the author. Supplementary material 00173-2026.SUPPLEMENT
