# Supplementary material for: Low-dose morphine and airway sensations in refractory chronic cough: a randomised control trial
Source: ERJ Open Res. 2026 Sep 1;12(5):00173-2026. doi: 10.1183/23120541.00173-2026 (PMC13531274; doi:10.1183/23120541.00173-2026)
Supplement: Supplementary file 1 [file 00173-2026.SUPPLEMENT.pdf]

Low dose morphine and airway sensations in refractory chronic cough: a randomised control trial

ONLINE DATA SUPPLEMENT

Bashar D Al-Sheklly[2], Joanne Mitchell[2], Kimberley Holt[1,2], Rachel Dockry[1,2], Huda Badri[1,2], Imran Satia[3], Terri Collier[2], Shilpi Sen[2], and Jaclyn A Smith[1,2]

[1]Division of Infection, Immunity and Respiratory Medicine, University of Manchester and Manchester Academic Health Science Centre; [2]Manchester University NHS Foundation Trust, Manchester, United Kingdom; [3]Department of Medicine, McMaster University, Canada

METHODS

Participants

Inclusion Criteria

- Male or Female >18 years old
- Diagnosis of refractory chronic cough (greater than 8 weeks duration) according to BTS guidelines
- Normal spirometry
- Treatment with low dose (5 to 10 mg) Morphine sulphate twice daily for more than 2 weeks and willing to temporarily withdraw treatment for study purposes

Exclusion Criteria

Patients meeting any of the following were excluded from participation in the study. Where criteria were time-dependent, patients may have been re-screened to enable inclusion:

- Current use of medications potentially modifying cough reflex sensitivity e.g. amitriptyline, pregabalin, codeine, etc.
- Participation in any clinical research study evaluating another investigational drug or therapy within 30 days or at least 5 half-lives (whichever is longer), of the investigational drug prior to consenting to study entry. If the subject was in an observational clinical study no washout was required
- History of medical conditions potentially modifying cough reflex sensitivity e.g. diabetes mellitus with autonomic neuropathy, Neurological conditions e.g. cerebrovascular disease, Parkinson's disease etc.
- Recent upper respiratory tract infection (<4 weeks)
- Pregnancy/breastfeeding

- Women of childbearing potential (not >2 years post-menopausal and/or not surgically sterilized) must have had a negative urine HCG pregnancy test, performed at visit 1 prior to enrolment into the study
- Current smokers or ex-smokers with <6 months abstinence or cumulative history of >20 pack years
- Current treatment with ACE inhibitors
- History of drug or alcohol abuse
- Uncontrolled hypertension (i.e., >160/90 mmHg despite adequate medical therapy).
- Recent myocardial infarction, or history of congestive cardiac failure
- Any clinically significant neurological disorder
- Any clinically significant or unstable medical or psychiatric condition that would interfere with the patient's ability to participate in the study

Procedures

Modified citric acid cough challenge; Volunteers were asked to inhale increasing concentrations of citric acid solution through a flow regulated air driven nebuliser. Doses of citric acid were prepared prior to the start of the study. This includes a total of 20 pots, 18 of them contained doses of citric acid. In all cases, 1 placebo dose of 0.9% saline at the beginning of the challenge was given to patients to have the chance to practise the test (pot 1). The test then started with an additional blind placebo pot (pot 2), also containing 0.9% saline. The 4 M stock solution was serially diluted in physiological saline to produce incremental concentrations of 0.01, 0.015, 0.02, 0.03, 0.04, 0.06, 0.09, 0.125, 0.18, 0.25, 0.375, 0.5, 0.75, 1.0, 1.5, 2, 3, 4M. Fresh dilutions of stock solution (Stockport Pharmaceuticals Ltd) were made on each day of testing.

State trait anxiety index; the state trait anxiety index (STAI) is a 40-point self-reported inventory developed by Spielberger et al(1). The aim is to identify one's current anxiety levels (state-scale) and their propensity to anxiety in general (trait-scale). Thus, two separate scores are given (state score and trait score), with higher values reflecting increased propensity to anxiety. Much work has been done to verify its validity and it has also been shown to be a sensitive predictor of caregiver distress, which can vary in response to changes in circumstances(2).

Somatosensory amplification scale; Barsky et al developed the somatosensory amplification scale (SSAS)(3). It is a 10 item self-report questionnaire, which asks the respondent how much they are affected by various visceral and somatic sensations. These sensations are not pathological symptoms of serious disease. It is a validated tool and was developed in a study that enrolled both healthy volunteers and those with an established diagnosis of hypochondriasis. Once again, higher numeric values reflect a greater propensity to somatosensory amplification.

The following were recorded on a 100mm VAS after each inhalation of citric acid:

- Intensity of throat tickling or itching
- Intensity of throat irritation
- Intensity of urge to cough
- How strong the sense of taste was
- Number of coughs evoked within 15 seconds (counted and documented by the researcher and verified by recordings on the VitaloJAK cough monitor).

The challenge stopped once the C2 i.e. concentration of citric acid inducing two coughs was reached or if volunteers completed all 20 inhalations without reaching C2.

Data Analysis

To identify whether coughing was associated with irritant airway sensations, the rate of change in sensations before coughing and at cough initiation, the C2, were compared. The rate of change

was measured in mm/mM reflecting changes in the VAS in mm per millimolar concentration of citric acid inhaled. The rate of change of each sensation at cough initiation was calculated as the VAS score at C2, minus the VAS score for the preceding concentration (C2-1); this was then divided by the difference in citric acid concentration between these points.

The rate of change of each sensation from the start of the challenge up to the dose just before coughing (C2-1) was then calculated. This was done by subtracting the VAS score at the first inhalation from that at C2-1. This was then divided by the difference in citric acid concentration between these two points.

A Wilcoxon test was then applied to assess whether the rate of change was significantly higher at C2 compared to the preceding inhalations.

RESULTS

Experimentally evoked sensations, SSAS and STAI scores

At C2 (the point of coughing at least twice in response to citric acid), VAS scores for throat irritation, tickle, urge to cough and taste did not correlate with SSAS scores, see Table E2.

At C2 (the point of coughing at least twice in response to citric acid), VAS scores for throat irritation, urge to cough and taste did not correlate with STAI-state scores at baseline, see table E2. A modest correlation was seen between tickle and STAI-state scores in this subgroup with an R value of 0.429, which proved to be statistically significant ($p=0.046$).

At C2 (the point of coughing at least twice in response to citric acid), VAS scores for throat irritation, tickle, urge to cough and taste did not correlate with STAI-trait scores at baseline (see table E2).

Experimentally evoked sensations and cough: effects of morphine therapy

When aligning sensation scores by the C2 dose it was found that morphine sulphate reduced estimated marginal mean VAS ratings for all 4 sensations compared to placebo. However, only urge to cough and taste reductions were statistically significant (both $p<0.001$, figure E2).

REFERENCES

1. Spielberger CD GR, Lushene R, Vagg PR, Jacobs GA. Manual for the State-Trait Anxiety Inventory. Palo Alto, CA: Consulting Psychologists Press, Inc, 1983.

2. Elliott TR, Shewchuk RM, Richards JS. Family caregiver social problem-solving abilities and adjustment during the initial year of the caregiving role. *Journal of Counseling Psychology* 2001; 48: 223-232.

3. Barsky AJ, Wyshak G, Klerman GL. The somatosensory amplification scale and its relationship to hypochondriasis. *J Psychiatr Res* 1990; 24: 323-334.

TABLES

Table E1 Wilcoxon test results for rates of change in sensations before and at C2 in RCC patients.

	Tickle pre cough vs C2	Irritation pre cough vs C2	Urge to Cough pre cough vs C2	Taste pre cough vs C2
**Z**	-2.983	-2.202	-3.662	-1.992
**p value**	**0.003***	**0.028***	**≤0.001***	**0.046***

Table E2 SSAS and STAI scores for RCC patients. Data are means and standard deviations.

**Questionnaire scores**	**Chronic Cough Patients (n=22)**
**Somatosensory amplification score (SSAS)**	25.2 (8.6)
**State trait anxiety index –State (STAI S)**	31.1 (9.4)
**State trait anxiety index -Trait (STAI T)**	34.7 (9.3)

Table E3 Correlation between scores of listed questionnaires and Visual analogue scales (VAS) at C2 for each sensation measured, in chronic cough patients. Data are correlation coefficient (r values) and p values using Spearman's analysis

Questionnaire	Sensation		Chronic Cough Patients
**Somatosensory Amplification scale (SSAS)**	**Tickle**	R value	-0.002
		P value	0.992
	**Irritation**	R value	-.188
		P value	.401
	**Urge to cough**	R value	-.008
		P value	.971
	**Taste**	R value	-.134
		P value	.551
**State trait anxiety index –State (STAI S)**	**Tickle**	R value	**.429**
		P value	**.046**
	**Irritation**	R value	.337
		P value	.125
	**Urge to cough**	R value	.420
		P value	.051
	**Taste**	R value	.001
		P value	.998
**State trait anxiety index –Trait (STAI T)**	**Tickle**	R value	.378
		P value	.083
	**Irritation**	R value	.207
		P value	.355
	**Urge to cough**	R value	.300
		P value	.175
	**Taste**	R value	.005
		P value	.983

FIGURES

Figure E1. Bland Altman plots within subject correlations for the logged VAS scores of UTC and tickle [A], irritation [B] and taste [C]with associated P and R values. Individual subject ID colour key can be seen on the right.

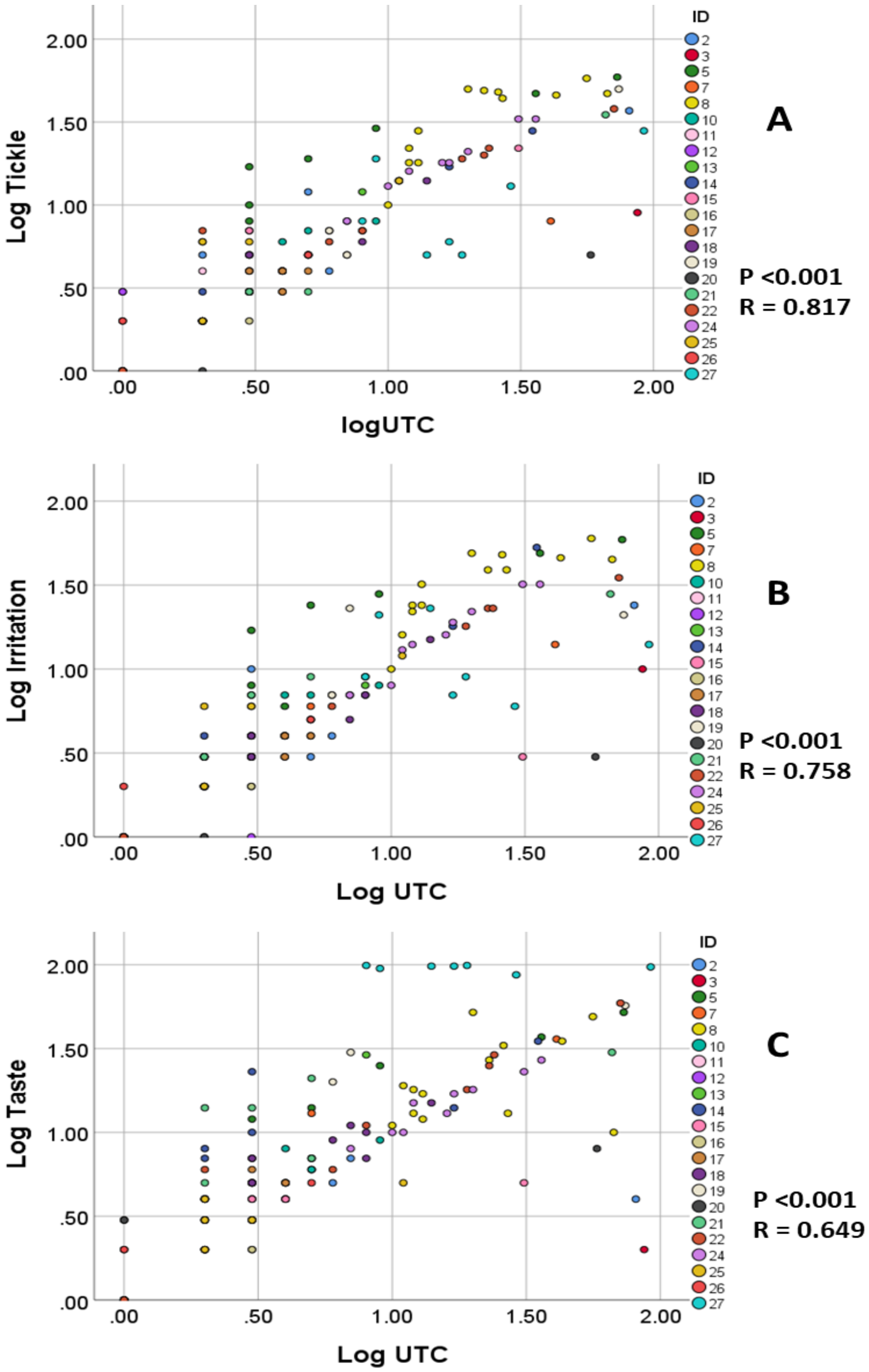

Figure E2

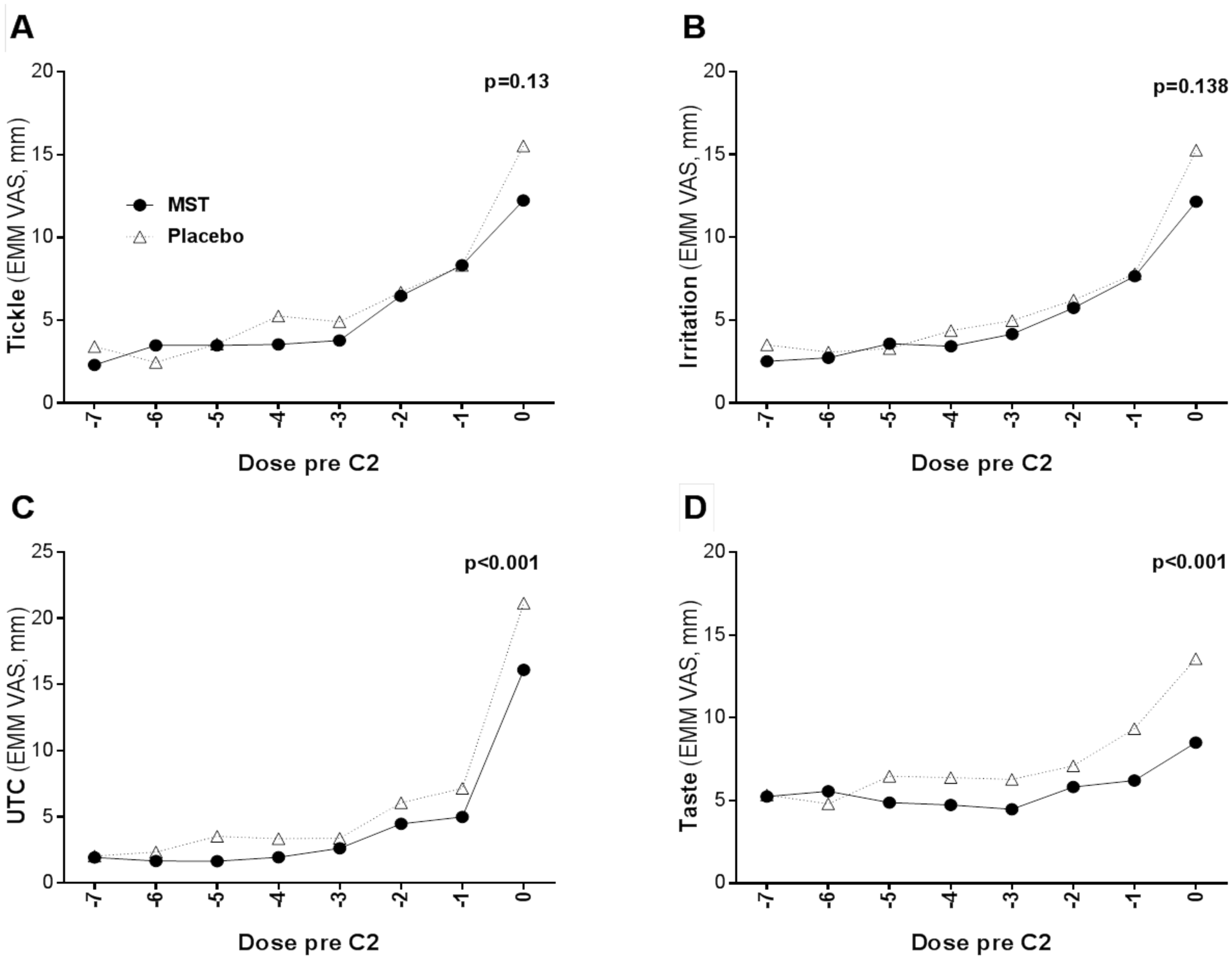
A
p=0.13
Tickle (EMM VAS, mm)
MST
Placebo
Dose pre C2
B
p=0.138
Irritation (EMM VAS, mm)
Dose pre C2
C
p<0.001
UTC (EMM VAS, mm)
Dose pre C2
D
p<0.001
Taste (EMM VAS, mm)
Dose pre C2

FIGURE E3

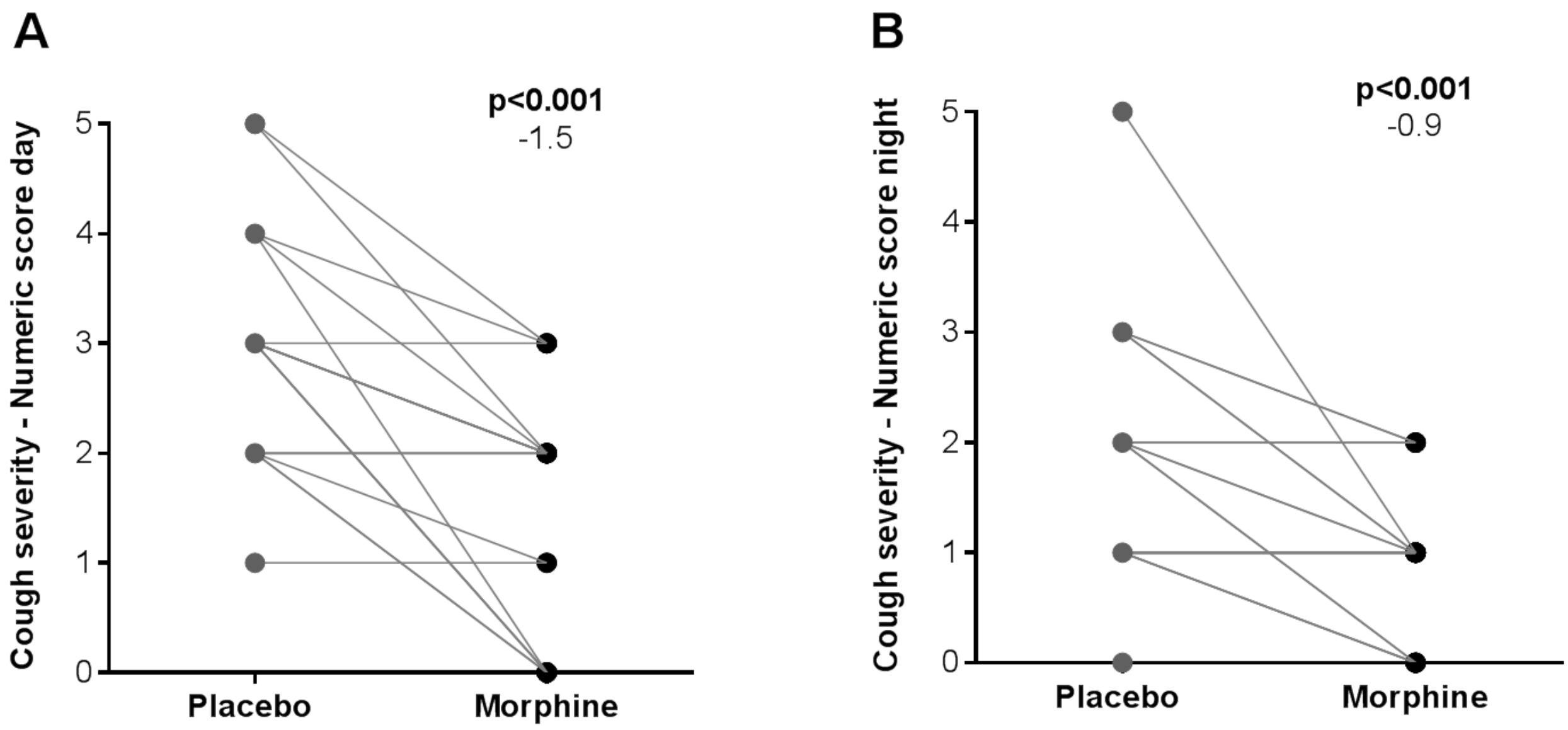
A
p<0.001
-1.5
Cough severity - Numeric score day
5
4
3
2
1
0
Placebo
Morphine
B
p<0.001
-0.9
Cough severity - Numeric score night
5
4
3
2
1
0
Placebo
Morphine

Cough Severity VAS

Memosa Cough Scoring

Study ID.. Date ....

Please indicate with a vertical line on the scale below your perception of the severity of your cough:

Day

|——————————————————————————|

No cough Worst Cough

Night

|——————————————————————————|

No cough Worst Cough

Please indicate your cough on the following scale for both day and night:

Day = ______________ Night = ________________

	Daytime	Overnight
0	No cough during day	No cough during night
1	Cough for one short period	Cough on waking only
2	Cough for more than two short periods	Wake once or early due to cough
3	Frequent coughing which did not interfere with usual daytime activities	Frequent waking due to cough
4	Frequent coughing which did interfere with usual daytime activities	Frequent coughs most of the night
5	Distressing cough most of the day	Distressing cough most of the night

Cough Challenge VAS

Study ID/ Patient No............................ Date ....

Visit No... CA Dose.................

Cough Challenge Visual Analogue Scales

1. Please rate the **tickling/ itching sensation** in your throat?

None strongest

2. Please rate the **irritation** in your throat?

None strongest

3. Please rate your **urge to cough**?

None strongest

4. Please rate the taste you experience?

None strongest
